# Topological Invariants of Vapor–Liquid, Vapor–Liquid–Liquid and Liquid–Liquid Phase Diagrams

**DOI:** 10.3390/e23121666

**Published:** 2021-12-10

**Authors:** Anastasia V. Frolkova

**Affiliations:** Lomonosov Institute of Fine Chemical Technologies, MIREA–Russian Technological University, 119454 Moscow, Russia; frolkova_nastya@mail.ru; Tel.: +7-916-665-8397

**Keywords:** phase diagram, vapor–liquid equilibrium, liquid–liquid equilibrium, topological invariant, Euler characteristic

## Abstract

The study of topological invariants of phase diagrams allows for the development of a qualitative theory of the processes being researched. Studies of the properties of objects in the same equivalence class may be carried out with the aim of predicting the properties of unexplored objects from this class, or predicting the behavior of a whole system. This paper describes a number of topological invariants in vapor–liquid, vapor–liquid–liquid and liquid–liquid equilibrium diagrams. The properties of some invariants are studied and illustrated. It is shown that the invariant of a diagram with a miscibility gap can be used to distinguish equivalence classes of phase diagrams, and that the balance equation of the singular-point indices, based on the Euler characteristic, may be used to analyze the binodal-surface structure of a quaternary system.

## 1. Introduction

In chemistry and chemical technology, both numerical and qualitative methods are actively used to study different phenomena and systems. The solution to a problem using numerical methods is a fixed result. The use of qualitative methods (dynamical systems theory, group theory, topology, set theory, etc.) allows for the study of the properties of models or objects in general and creates various theories with predictive capabilities. The theory of equilibrium in heterogeneous systems, based on the invariant representation of the Gibbs zero potential, was created using this approach. It has been successfully developed in the research of A.V. Storonkin [1] and his successors. The application of basic concepts of topology and a series of invariants made it possible for thermodynamic–topological analysis (TTA) to develop as the main, and most effective, method for studying phase diagrams of multicomponent systems (the founders of the method are V.T. Zharov, Yu.V. Gurikov, L.A. Serafimov [2,3,4,5,6,7]). This method is now widely used all over the world for studying complex systems with different phase behavior (liquid–vapor equilibrium, liquid–liquid, liquid–liquid–vapor, liquid–solid, etc.) including those with a chemical reaction [8,9] and mass-transfer processes. A detailed analysis of the work in this area is given in the review, [10]. The development of TTA entails: identification of certain topological features, in particular invariants of phase diagrams, in order to describe and predict the properties of a set of objects, for example, belonging to the same class; obtaining a complete set of diagrams and developing their equivalence classification; and, finally, studying aspects of diagram evolution. Such topological invariants include: the Euler characteristic, surface genus, connectivity, type and Poincaré index of a singular point. Topological properties of phase diagrams are considered in the literature [2,3,4,5,6,7,11,12,13,14,15,16,17,18,19,20,21,22,23,24,25]: vapor–liquid equilibrium [2,3,4,5,6,7,11,12], liquid–liquid equilibrium [13,14,15] and liquid–solid equilibrium (LSE) [16,17,18,19,20,21,22,23,24].

In the present paper, the main invariants of vapor–liquid (VLE), liquid–liquid (LLE) and vapor–liquid–liquid (VLLE) equilibrium diagrams are considered. The aim of this work is to study the properties of topological invariants, determine the scope of their applicability and illustrate of their possible use for the development of equivalence classifications. The properties of the integral Poincaré invariant, the invariant of the diagram with a miscibility gap, are studied. The objects of theoretical research are phase diagrams and their internal geometric elements (separatrix (VLE) and binodal (VLLE) surfaces). The material is based on analysis of the literature of Professor L.A. Serafimov’s scientific group, as well as on results of the author’s research.

### Topological Concepts Considered

The basic terms and formulas used in the description of topological invariants of phase diagrams and their constituents are given below.

Euler characteristic for closed manifolds (provided that the genus of the surface is zero) [2,11]:

(1) $$E=1+{\left(-1\right)}^{m},$$

where *E* is the Euler characteristic and *m* is sphere dimension.

Euler characteristic for a manifold with boundary [2,11]:

(2) $$E=\alpha_{0}-\alpha_{1}+\alpha_{2}-\ldots -{\left(-1\right)}^{m}\alpha_{m},$$

where $\alpha_{m}$ is an element of a manifold of dimension *m*. Elements of even dimension are included in Equation (2) with a plus, while elements of odd dimension, with a minus. A manifold with boundary and its elements are, together, an open set.

The VLE diagram is a graphical representation of a system of differential equations describing the distillation process [2]:

(3) $$\frac{dX}{d\tau }=\bar{Y-X},$$

where dX is the vector of liquid-phase composition change; $\bar{Y-X}$ is the liquid–vapor node vector; dτ=dlnM, where *M* is the mass of liquid.

Equation (3) is nonlinear and its direct integration is impossible. To solve a system of nonlinear differential equations, methods of qualitative analysis of the equations around singular points ($\frac{dX}{d\tau }=0$) are used. These points are pure components and azeotropes. Qualitative analysis involves the linearization of Equation (3) around a singular point through Taylor series expansion. At the same time, the concept of characteristic roots (*λ*) of the matrix of coefficients, of equations in a linearized system, is introduced. The dynamic system in question implements only singular points of the saddle (*S*: roots of different signs) and node (*N*: all roots of the same sign) type. The number of roots is *n* − 1 (*n* is the number of components in the system) [2,4,5,6].

The Poincaré index of singular points can be determined from the formula [2,4,5,6]:

(4) $$sign\;i=sign\;\overset{n-1}{\underset{1}{\prod }}\lambda_{j}$$

The Poincaré index is applicable to points that have a complete neighborhood.

Any residue-curve diagram can be correlated with an isothermal–isobar diagram, in which singular points of the node type are represented by elliptical-type points, and saddles by hyperbolic-type points [2,4,5,6].

The Poincaré integral invariant is an integral that remains constant when the integration domain moves under the flow action [1,2,3]. The motion of a point in a space of *l* dimension is described by a system of ordinary differential equations. The variety of points occupying an *l*-dimensional region at the initial moment will occupy a *k*-dimensional region at any subsequent moment, provided that *k* < *l*. The *k*-fold integral extended to a given domain is called the Poincaré integral invariant [12,26,27].

## 2. Results

### 2.1. Vapor–Liquid Equilibrium and Vapor–Liquid–Liquid Equilibrium

#### 2.1.1. Euler Characteristic and the Balance Rule for Singular-Point Indices

The first topological invariant considered is the Euler characteristic, and the balance rule of the indices of singular points based on it. This rule has the character of a law. In general, the following formulation can be given: “the algebraic sum of singular points, taking into account the sign of their index of any vector field placed on a sphere, is equal to the Euler characteristic of the sphere (1)” [2,11].

Two forms of the azeotropy rule are known. The difference is in the method of constructing a dihedron, and its subsequent mapping to a sphere. The first one is proposed in [2,3]. The formula of the azeotropy rule in general is as follows [1]:

(5) $$\sum 2^{m+1}\left(N_{i}^{+}+S_{i}^{+}-N_{i}^{-}-S_{i}^{-}\right)=E,$$

where *E* is the Euler characteristic determined by Equation (1), *m* is the dimension of the element on which the singular point is located, “+” and “−” correspond to the sign of the Poincaré index of the singular point of saddle (*S*) or node (*N*) type.

The second rule presented in [2,28] has the form:

(6) $$2\left(N_{m}^{+}+S_{m}^{+}-N_{m}^{-}-S_{m}^{-}\right)+\overset{m-1}{\underset{0}{\sum }}\left(N_{i}^{+}+S_{i}^{+}-N_{i}^{-}-S_{i}^{-}\right)=E,$$

where *m* (*i*) is the dimension of the element on which the singular point is located (in this equation, *m* refers to the inner space, *i* refers to the boundary space)

A detailed analysis of both equations is given in [11,29,30]. It should be noted that the equation in the form (5) has limitations in its application; in particular, it cannot be used for non-simplicial structures [30]. Another difference between the two approaches is the analysis of the phase diagram boundary space. According to L.A. Serafimov, complex singular points with a zero Poincaré index may be present on the boundary space of the diagram. This approach is convenient because it allows identification of saddle-type singular points, for which only one of the roots (*λ*) takes a positive (negative) value and the remaining roots are less (greater) than zero, at the stage of analyzing the boundary space structure (as a rule, already at the stage of analyzing the second dimension scan). These points generate separatrix (hyper) surfaces, dividing a composition simplex on the distillation region [31,32].

Regardless of which form of the equation is chosen, the sum of the indices of singular points for VLE diagrams of even dimension will always be equal to two; for odd dimension, it is zero.

Based on the solution of the azeotropy rule, all possible structures of VLE diagrams of the ternary systems were obtained, and their equivalence classifications were proposed [2,3,33].

**Figure 1:**
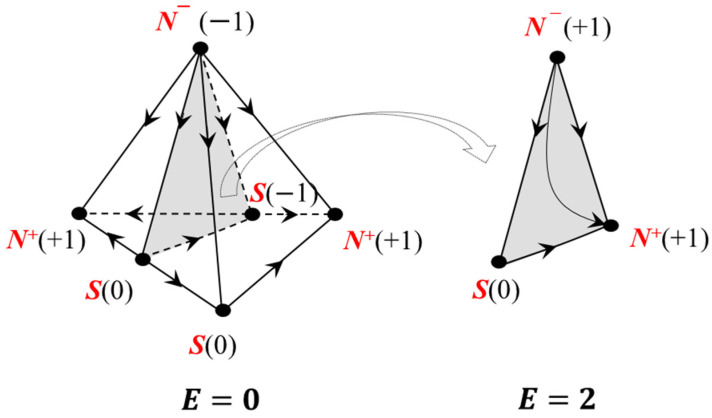
The structure of the VLE diagram and the separatrix surface generated by the azeotrope of saddle type (C): point types are indicated in red; Poincaré indices are shown in parentheses. The verification of the azeotropy rule is given for the formula of L.A. Serafimov.

The azeotropy rule can be applied not only to the complete structure of the VLE diagram of an *n*-component system or its scan of (*n* − 2) dimension, but also to its fragments, for example, the separatrix (hyper)surfaces (Figure 1).

**Figure 2:**
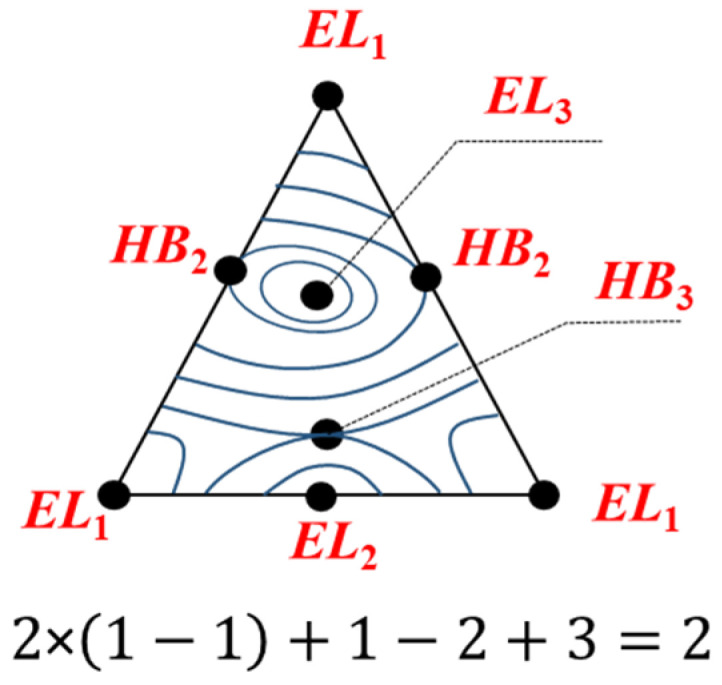
An example of a scalar-property isolines diagram and an illustration of the feasibility of Equation (7).

To study the scalar properties (temperature, pressure, relative volatility, heat capacity, etc.) of ternary systems, isoline diagrams, which are characterized by the presence of their own singular points of elliptical, hyperbolic type, are often used (note that parabolic type occurs for the ternary-system diagrams only in the bifurcation state [34]). An equation that is an analogue of the azeotropy rule can be written for such diagrams [35]:

(7) $$2\left(EL_{3}-HB_{3}\right)+EL_{2}-HB_{2}+EL_{1}=E$$

where *EL* (*HB*) is the point of elliptical (hyperbolic) type. An example is given in Figure 2.

**Figure 3:**
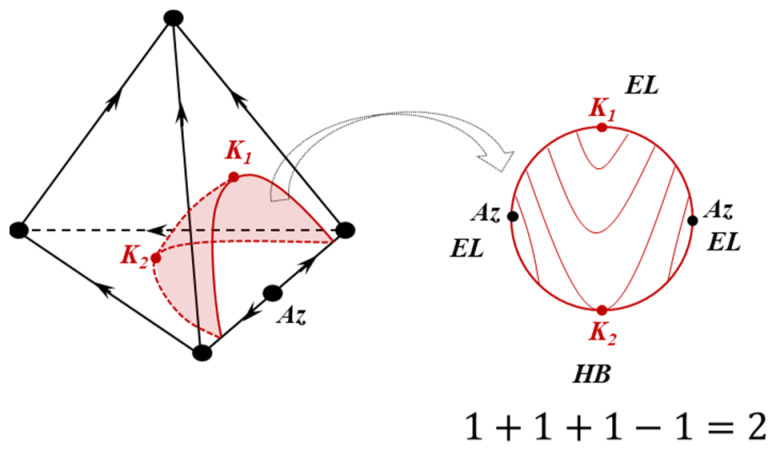
The structure of a VLLE diagram of a quaternary system, isotherms–isobars of binodal surface and an illustration of the feasibility of Equation (7). *K*_1_, *K*_2_: critical points (the boiling point for *K*_1_ is higher than for *K*_2_); *Az*: azeotrope.

Equation (7) can be used to analyze the structure of diagram fragments, in particular, the binodal manifold (at P = const) of quaternary systems (VLLE is considered). Singular points of such a surface are the points of equilibrium of liquid layers corresponding to azeotropes, as well as critical points. The binodal manifold can be covered with lines of a constant boiling point (isotherm–isobar lines) and each singular point will be presented as an elliptic or hyperbolic point. An example of a phase diagram and a binodal surface is shown in Figure 3. Thus, Equation (7) is fulfilled.

#### 2.1.2. The Balance of Singular-Point Indices Participating in the Bifurcation

**Figure 4:**
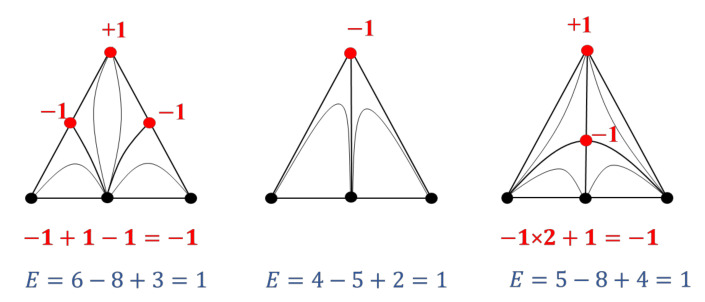
Example of a ternary-system diagram bifurcation [2] (distillation lines are not oriented, the points participating in the bifurcation are highlighted in red).

Separately, the balance of singular-point indices participating in the bifurcation can be distinguished: “the sum of the indices of the singular points participating in the bifurcation is a constant value”. Figure 4 shows an example of a bifurcation diagram of a ternary system [2]. The points involved in the bifurcation as well as the values of their Poincaré indices are highlighted in red. The sum of the topological indices of the singular points in the ternary-system diagram before and after bifurcation are equal.

#### 2.1.3. The Euler Characteristic as an Alternative Sum of Elements of Different Dimensions

**Figure 5:**
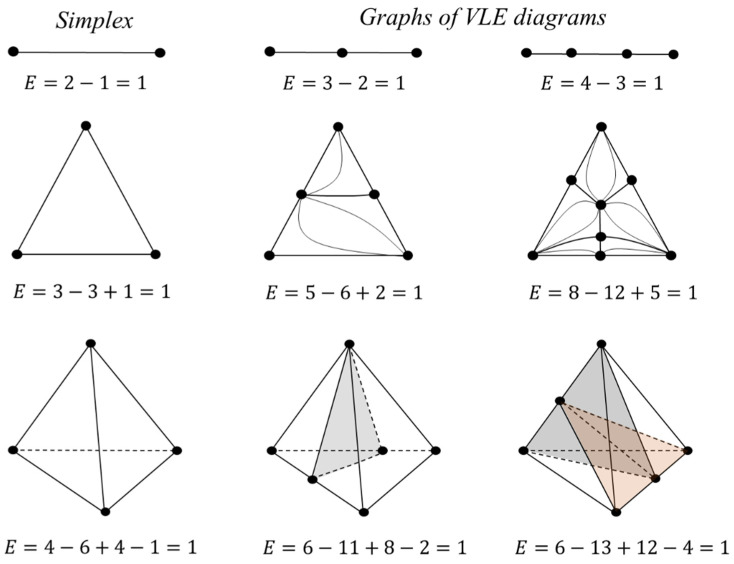
Euler characteristic for simplexes and graphs of VLE diagrams for systems with different numbers of components.

Graph theory is often used to study the structure of VLE diagrams when the constituents of the diagram, its singular points (pure components and azeotropes) and manifolds (separatrix (hyper) surfaces), correspond to the vertices, edges, faces and hyperfaces of the graph. The elements of different dimensions of the graph are interconnected in Equation (2). Each element is a manifold without boundary [36]. For such manifolds, the property is characteristic: if a space of *m* dimension is divided into parts by spaces one dimension smaller, then the Euler characteristic will not change, i.e., it is a topological invariant. This is illustrated with VLE diagrams of systems with different numbers of components (2–4) (Figure 5). The phase diagram is characterized by a simplicial structure. For any simplex, the Euler characteristic (determined by Formula (2)) is always equal to 1. Regardless of how many manifolds of different dimensions are present in the system (azeotropes, separatrix surfaces), the Euler characteristic of the diagram will not change [30]. This value of *E* is also preserved during the bifurcation of the VLE diagrams through intermediate structures containing tangential azeotropes (Figure 4, calculation of *E* according to (2) is highlighted in blue).

**Figure 6:**
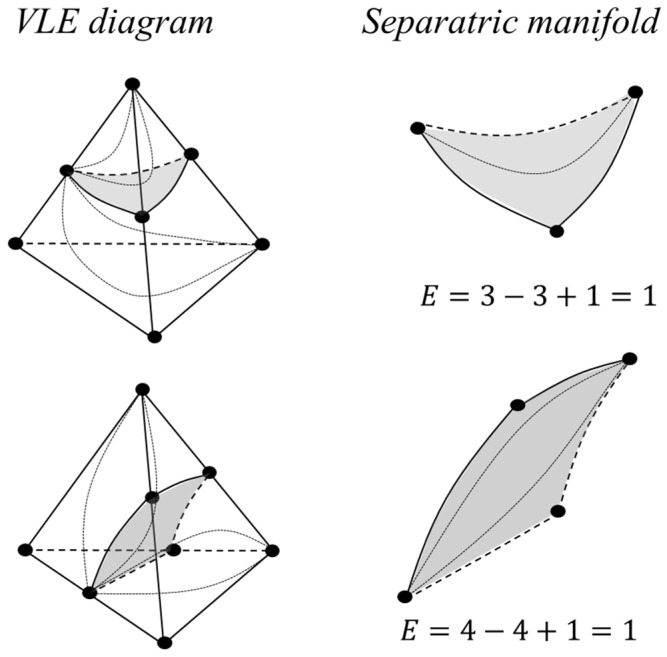
Euler characteristic for separatrix surfaces of simplicial and non-simplicial structures.

It is possible to consider fragments of the phase diagram, for example, separatrix manifolds (both simplicial and non-simplicial structures), and make sure that this invariant is also characteristic of them (Figure 6).

For the boundary space of the VLE diagram, Equation (2) takes the form:

(8) $$E=\alpha_{0}-\alpha_{1}+\alpha_{2}-\ldots -{\left(-1\right)}^{m-1}\alpha_{m-1},$$

If we consider that the diagram boundary space of an *n*-component system is homeomorphic to the sphere of dimension *n* − 2, the Euler characteristic can be determined by Equation (1) (*m* = *n* − 2). As an example, let us consider the boundary space of a ternary zeotropic system, which consists of three vertices and three edges. The value of the Euler characteristic can be determined by Formula (8): *E* = 3 − 3 = 0. Conversely, the boundary of a triangle is homeomorphic to a one-dimensional sphere for which, according to Formula (1), *E* is also zero.

#### 2.1.4. Poincaré Integral Invariant and Separatrix Manifolds

The authors of [12] correlated the process of distillation with the phase process described above, i.e., the motion of points in space that were considered by Poincaré [26] (the Cauchy-uniqueness principle was implemented for both processes). Analysis of the VLE diagrams of systems with more than three components, in particular the internal separatrix surfaces contained in them, showed that the latter have properties in common with the Poincaré integral invariant, namely, the presence of a subspace of a smaller dimension in *l*-dimensional space, the presence of a certain structure of the subspace under consideration and its closure.

**Figure 7:**
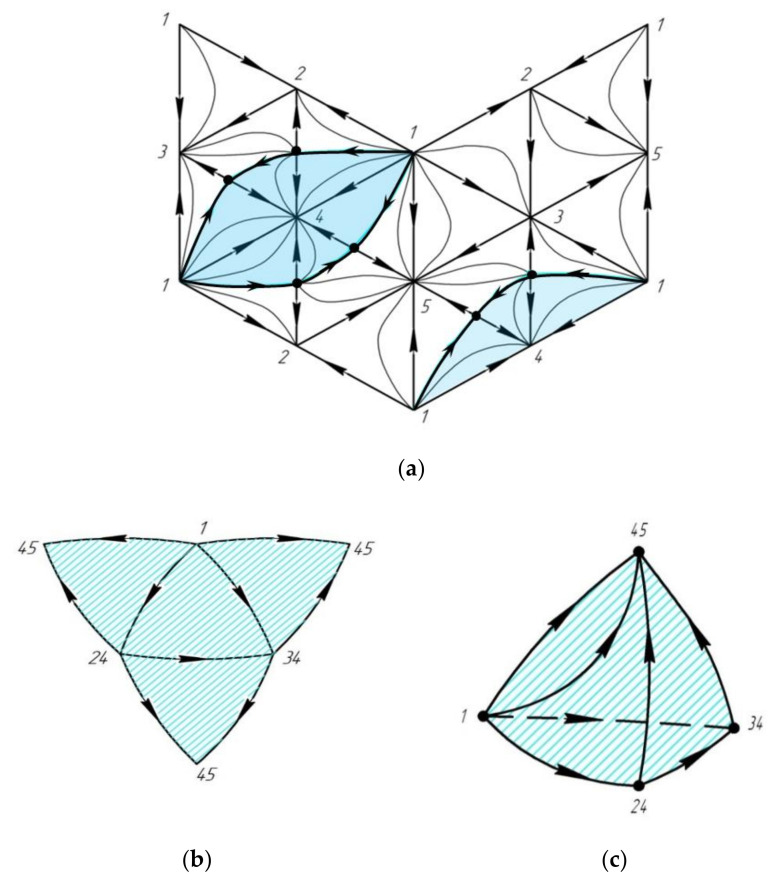
(**a**) Two-dimensional scan of the VLE diagram of a five-component system; (**b**) scan and (**c**) complete structure of a three-dimensional separatrix manifold.

Figure 7a shows a two-dimensional scan of a five-component system. There is one saddle point in the system with a non-zero Poincaré index (this point is not a complex one with respect to the boundary space): azeotrope 45 (according to L.A. Serafimov’s approach). Consequently, the composition pentatope contains a three-dimensional separatrix hypersurface that divides it into two four-dimensional distillation regions [32].

It can be seen from Figure 7 that a one-dimensional closed line is formed on the boundary space (highlighted with a bold black line), which is the one-dimensional boundary of the separatrix hypersurface. A fragment of the three-dimensional scan of the pentatope is shown in Figure 7b; it corresponds to the boundary space of the separatrix manifold (it also forms a closed contour). The structure of the considered hypersurface is given in Figure 7c. It is topologically similar to the VLE diagram of a zeotropic quaternary system. This third-dimension hypersurface is a closed manifold characterized by its own structure and is located in a composition space of dimension four, i.e., it has all the properties of the integral Poincaré invariant.

If we analyze one-dimensional separatrices of ternary systems, we can see that some of the listed properties are not characteristic of them. This suggests that ternary systems cannot be fully considered as multicomponent, but rather as an intermediate link between binary systems and systems with over three components (multicomponent). The analysis of the boundary space of multicomponent systems always begins with a two-dimensional scan. It is already possible to predict the structure of the complete diagram at this stage of the study: to identify singular points of the saddle type, generating separatrix (hyper) surfaces of (*n* − 2)-dimension and the number of distillation regions. For ternary systems, the scan of the phase diagram is one-dimensional, i.e., it is impossible to determine the presence of internal separatrices only on the basis of its analysis.

Thus, separatrix manifolds of dimension two and higher can be distinguished into a special group of VLE diagram elements that have the properties of the integral Poincaré invariant [26].

The balance equation of the singular-points indices, as well as the above-described properties of separatrix (hyper) surfaces, were the basis for developing a methodology for studying the internal space of VLE diagrams of multicomponent systems. The existence of two ternary azeotropes in the benzene + perfluorobenzene + water system was predicted and confirmed experimentally [37]; five new quaternary azeotropes [38], and one five-component [32] azeotrope were found using this technique.

### 2.2. Liquid–Liquid Equilibrium

#### 2.2.1. Invariant of the LLE Diagram

The boundary space of an *m*-dimensional phase diagram is homeomorphic to a sphere of dimension (*m* − 1): a one-dimensional sphere corresponds to the boundary of a triangle; a two-dimensional, to the boundary of a tetrahedron; a three-dimensional, to the boundary of a pentatope; etc. The Euler characteristic for the boundary space is determined, as previously mentioned, by Formula (8); for a sphere, using Formula (1), these values coincide. If a “hole” appears on a sphere of a certain dimension, then its Euler characteristic will decrease by one [11,14].

**Figure 8:**
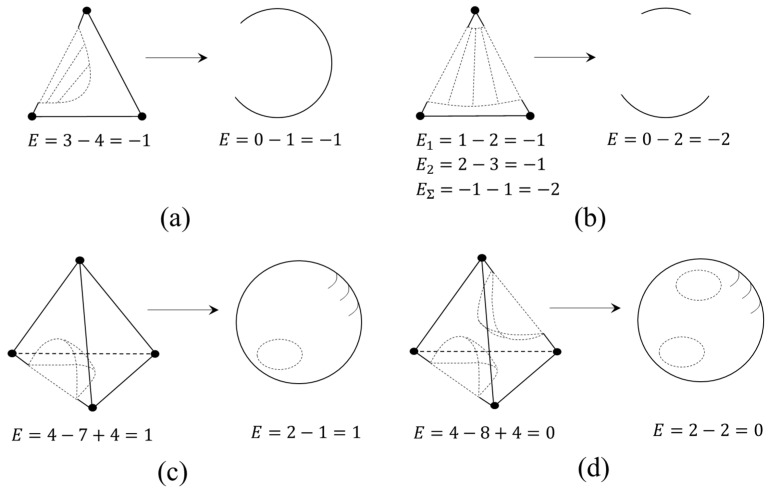
Mapping the boundary space of the diagram a with miscibility gap onto a sphere: ternary system with one (**a**) and two (**b**) liquid-phase splitting binary constituents; quaternary system with one (**c**) and two (**d**) liquid-phase splitting binary constituents.

Figure 8 shows examples of diagrams with miscibility gaps of ternary and quaternary systems. Let us perform the following actions: the miscibility area is “cut out” from the diagram. When mapping the remaining boundary space onto a sphere, one can see that a “hole” is formed on the latter. At the same time, regardless of the chosen method for calculating the Euler characteristic (Formula (1) or Formula (8)), it remains unchanged and its value decreases by the number of “holes” on the sphere.

Thus, we can write the following formula for the boundary space of the phase diagram:

(9) $$E=\alpha_{0}-\alpha_{1}+\alpha_{2}-\alpha_{3}+\ldots =\left(1-{\left(-1\right)}^{n-2}\right)-d,$$

where *d* is the number of “holes” on the sphere. The resulting equation is an invariant of the LLE diagram describing its boundary space. It is also important to note that the additive properties of the Euler characteristic are also preserved with this approach, which is illustrated by the example of a ternary system with an open liquid-phase splitting region (without a critical point) (Figure 8b): *E*_1_ and *E*_2_ are the Euler characteristics of the upper and lower parts of the boundary space of the diagram, and *EΣ* is the sum of *E*_1_ and *E*_2_.

**Figure 9:**
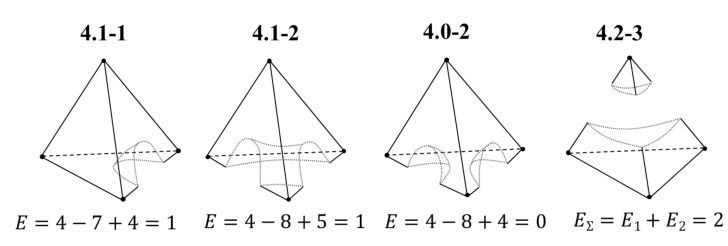
Example of classification of LLE diagrams based on their invariants.

This invariant can also form the basis for the equivalence classification of LLE diagrams. Diagrams of *n*-component systems with the same Euler characteristic can be allocated to one class; the types of diagrams within the same class will differ in the number of liquid-phase splitting binary constituents. As an example, let us consider class 4.1, where 4 is the number of components in the system and 1 is the value of the Euler characteristic. Within this class, two types can be distinguished: 4.1-1 (the system contains one liquid-phase splitting binary constituent) and 4.1-2 (the system contains two liquid-phase splitting binary constituents) (Figure 9).

#### 2.2.2. Invariant of the Liquid-Phase Splitting Region

Questions related to the number and choice of independent variables (the number of degrees of freedom) are of fundamental importance in studying heterogeneous systems. In general, the number of independent variables is equal to the difference between the number of all variables, and the number of independent equations connecting them. For open, *n*-component, *φ*-phase, chemically inert systems, the number of degrees of freedom the system (variation) is determined by the Gibbs phase rule:

(10) f=n−φ+2

The maximum number of liquid phases ($\varphi^{L}$) in the system is equal to the number of components, i.e., $\varphi =\varphi^{L}+1$ (1–corresponds to vapor phase). The phase simplex, whose vertices are the compositions of equilibrium liquid phases, is always linear. In particular, in the presence of two liquid phases, the liquid-phase splitting simplex is represented by a straight line (first dimension), in the presence of three liquid phases by an irregular triangle (second dimension), etc. The dimension of the phase simplex (*R*) with the maximum number of vertices *n* coincides with the dimension of the composition simplex and is generally equal to:

(11) $$R=\varphi^{L}-1$$

Suppose there is an *n* − 1 liquid phases in a particular liquid-phase splitting region of an *n*-component system. Then, the dimension of the phase simplex would be equal to *n* − 2, and the variation of the region would be 1. For the number of phases *n* − 2, the phase simplex is of dimension *n* − 3 and the variation in the region 2. Further, this trend of changing values is maintained. The number of liquid phases in the system under study is always one less than the number of phases (all liquid phases + vapor phase), and the dimension of the phase simplex is always one less than the number of liquid phases.

Substituting Equation (11) into the Gibbs phase rule (10) under the condition P = const or T = const (one variable is fixed), we can obtain [13]:

(12) R⊕f=n−1=const

For a mixture with a given number of components, the sum of the variation in the liquid-phase splitting region and the phase simplex dimension is a constant value.

**Figure 10:**
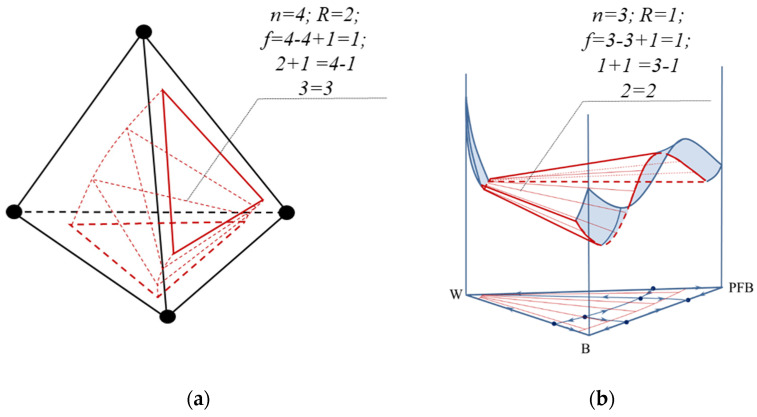
Fragment of the LLLE region in the diagram of a quaternary system: (**a**) the temperature surface of a ternary system of benzene (B) + perfluorobenzene (PFB) + water (W) [37]; (**b**) an illustration of the invariant of a liquid-phase splitting region.

Thus, Equation (12) defines the invariant of the liquid-phase region of a multiphase system. Since the number of external independent variables describing the cross section of the diagram of the heterogeneous system “complex of liquid phases”, the vapor phase, is always equal to *n* − 1 and does not depend on the number of phases, any liquid-phase splitting region can be represented as a direct sum of two manifolds, one of which is the phase simplex of the liquid state of dimension *R* (the linear constituent of the sum), and the other is a manifold whose dimension is equal to the variant *f* (the nonlinear constituent of the sum) (Figure 10a). Thus, the sum of the dimensions of the constituents of the splitting region is always equal to the dimension of the composition simplex *n* − 1. This invariant was the basis for developing methods for studying three liquid-phase splitting regions of quaternary systems [39].

The relationship between the direct sum of the linear and nonlinear constituents of the invariant can be demonstrated in the example of the temperature surface of a benzene + perfluorobenzene + water system [37] (a linear surface corresponding to a region of two liquid-phase splittings is highlighted in red). The minimum and maximum of this surface correspond to the node- and saddle-type ternary azeotropes, respectively. The uniqueness of this system is that it contains two ternary azeotropes and two binary azeotropes in the benzene + perfluorobenzene constituent.

## 3. Conclusions

This paper presented an overview of invariants for vapor–liquid-equilibrium, vapor–liquid–liquid-equilibrium and liquid–liquid-equilibrium diagrams. The properties of invariants were described, their roles in the study of the phase-diagram structure were shown and examples of their application were illustrated. Universal application of the singular-points-indices balance equation, based on use of the Euler characteristic, was shown to be possible for various object types. It was also confirmed by applying the balance equation to analysis of liquid–solid-equilibrium diagrams [20,21,22]. The difference of liquid–solid-equilibrium diagrams from VLE diagrams was in the greater variety of singular points in the latter. Using the balance equation to analyze the structure of isothermal–isobar binodal surfaces as geometric elements of the liquid-phase splitting diagram at constant pressure was demonstrated. All thermodynamically possible structures could be obtained by solving this equation.

Based on analysis of the properties of the integral Poincaré invariant, the difference between ternary systems and multicomponent systems (systems with more than three components) was shown. This difference was primarily due to the variety of singular points, to the feature of the boundary analysis and to the interior space of the diagram. This fact may indicate that, when studying various processes (for example, distillation) in multicomponent systems, consideration should not be limited to only ternary systems; it is necessary to involve systems with more than three components in the analysis.

To describe the properties of a phase diagram, it is necessary to abstract from its geometric structure and use a topological representation. Selection of phase-diagram invariants allows for the creation of equivalence classifications based on them. Several geometric structures can correspond to one topological image. The possibility of creating an equivalence classification using the invariant of the LLE diagram was illustrated in the paper.
